# Causes of Tinnitus in Patients Referred to ENT Clinic of Imam Khomeini Hospital in Urmia, 2012-2013

**DOI:** 10.5539/gjhs.v6n7p136

**Published:** 2014-09-18

**Authors:** R. Samarei, N. Fatholahi

**Affiliations:** 1 Head & Neck Surgeon, Urmia University of Medical Sciences, Urmia, Iran; 2 Urmia University of Medical Sciences, Urmia, Iran

**Keywords:** tinnitus, noise, acoustic trauma, ototoxicity, presbycusis

## Abstract

**Introduction:**

Tinnitus includes all the sounds perceived by the patient, without any external stimulus that affects all aspects of life; there is no cure for most patients. Given that tinnitus and hearing of the ear are common complaints of patients in clinics and due to the impossibility of cure at the present time, it is necessary to set up common causes of tinnitus to solve social problems and present optimal solutions. The purpose of this study was to determine the causes of tinnitus in patients referred to ENT Clinic of Imam Khomeini Hospital in Urmia, 2012-2013.

**Materials and Methods:**

This is a cross-sectional analytic study that was performed on 184 patients with tinnitus referred to ENT clinic of Imam Khomeini Hospital, Urmia. After examination, audiometry performed and questionnaires of tinnitus patients were completed. The results were analyzed by statistical SPSS software.

**Results:**

The study included 111 males and 73 females and the mean age was 50.6 years. 168 patients (91.3%) had non-pulsatile tinnitus and the rest had pulsatile tinnitus. The mean hearing loss in patients was 31.4 dB, 79.9% were of sensory neurons kind, and 12.5% of patients did not show any hearing loss in audiometry. In total, 90.2% of the cases were detected; most causes of tinnitus were noise (19.6%), ototoxicity (16.8%) and presbycusis (16.3%). The most common causes of tinnitus were noise with 31.5% in males and ototoxicity with 27.4% in females. Between age and hearing loss of patients, there was a significant relationship (0.001> P-value), but there was no significant relationship between gender and degree of hearing loss.

**Discussion:**

The most common cause of tinnitus (NIHL) is quite predictable, except by accident, you can completely avoid by reducing noise levels on the source and ear protection. By reasonable prescription of ototoxic medications particularly antibiotics can reduce the prevalence of tinnitus that causes sleep disorders and concentration problems, depression and anxiety and help the public health.

## 1. Introduction

Tinnitus includes all the sounds perceived by the patient, without any external stimulus (Cummings et al., 2005). The sound is a symptom of an underlying problem in the hearing way that shows, tinnitus is a symptom similar to pain and like pain is created after an environmental injury (Bauer & Brozoski, 2008). Tinnitus is one of the three main symptoms of ear diseases or disorders of hearing and balance, sometimes the symptoms are so distressing that overshadow the hearing of person and the person does not complain of hearing loss but the buzz is much complaining (Madani Syed Abdullah Mohammad Kebri, 2001). Buzz, has an overall impact on the patient’s life and the negative impact on quality of life in 2% of the adult population of the whole world (Mazurek et al., 2010). These effects include mental health problems, hearing, sleep and focus. There are also reports stating that tinnitus can cause fear, unpacking, anger, irritability and anxiety (Durch, Humes, & Joellenbeck, 2005). Since the beginning of human civilization man is suffered from tinnitus and at the present time the number and severity of tinnitus sufferers have been added (Chen et al., 2013). The first known handwritten in introduction and treatment of tinnitus has been seen in the 16th century BC in Egyptian papyrus that shows, human history is a story of greatness (Madani Syed Abdullah Mohammad Kebri, 2001). First epidemiologic study concerning tinnitus was conducted by Mr. Hinchcliffe in 1961; in this study, the prevalence of tinnitus from 21% in the age group of 24-18 years to 39% in the age group of 64-55 years has been variable (Madani Syed Abdullah Mohammad Kebri, 2001). Tinnitus prevalence depending on the studied population varies from 3% to 30% and its intensity varies from almost indestructible feel to severe and debilitating (Cummings et al., 2005). Approximately fifty million people in America have experienced tinnitus and of those, only 16 million people have persistent tinnitus. Prevalence of continuous tinnitus increases with age, so that its peak is in the seventh decade of life (14.3%) (Shargorodsky, Curhan, & Farwell, 2006)

In general, in most of the books tinnitus is classified as either subjective or objective. Objective tinnitus can be heard by the patient and the person who examines, whereas subjective tinnitus can be heard only by the patient, which makes it difficult to assess; in addition, causes of subjective tinnitus is less known and there is no cure for most patients with chronic tinnitus (Cummings et al., 2005). Tinnitus is caused because of denial of nerve entry or unusual entry from ear. This leads to structural changes or functional alterations in neural input to the central nervous system, causing increased sensitivity or increased neural activity (Cummings et al., 2005). Subjective tinnitus is usually associated with hearing pathology and measurable impairment of hearing function. Two main causes for subjective chronic tinnitus are NIHL (Noise Induced hearing Loss) and presbycusis. It refers to a pathological cause of the auditory system as a source of tinnitus, but about 10% of subjective chronic tinnitus occurs in the absence of specific auditory pathology in routine clinical tests and 20% of those with profound hearing loss have not tinnitus (Bauer & Brozoski, 2008). Tinnitus is also, based on the sound quality felt by the patient, divided into pulsating and non-pulsating. Pulsatile tinnitus refers to the vascular causes, which can be subjective or objective, especially when the sound is synchronous with the arterial pulse, but if is not simultaneous with heart rate suggests non-vascular causes such as myoclonus com. Non-pulsatile tinnitus, which is a very common type of tinnitus is usually purely subjective; that in many cases associated with hearing loss that in these cases symptoms are attributed to the damage caused by noise and ototoxic drugs. Noise and ototoxic drugs cause direct damage to the hair cells, although the exact pathophysiology is not completely understood (Cummings et al., 2005).

Trauma (head injury, NIHL and acoustic trauma), presbycusis, Meniere’s disease, ear infections, neurological disorders (MS), musculoskeletal disorders (otosclerosis) and neoplasms (schwannomas and stybolar) are other causes of SNHL (Sensory Neural Hearing Loss) and are non -pulsatile tinnitus. Causes of pulsatile tinnitus include increased benign intracranial pressure, glomus tumor, atherosclerotic disease of the carotid artery, malformations and arterial venous fistulas of the head and neck, Eustachian tube dysfunction and etc. (Cummings, 2005). Infection of the outer ear, middle ear effusion and compact cerumen and can cause tinnitus with conductive hearing loss (Crummer & Hassan, 2004). The fact that exposure to loud noise can cause hearing loss, first became known in the 18th century and in 20th century, NIHL, Carey was called boiler manufacturers (Cummings, 2005). Many cross-sectional studies in society, industry and soldiers have shown that buzz in cases where there is long-term exposure to occupational noise are more likely to be seen; many studies have shown the prevalence of tinnitus with some degree of hearing loss is more. A study in Spain in 2007 showed that 100% of people who worked in noisy environments had hearing impairment in audiometry (Proupin Vazquez et al., 2007). In a study in 1995 on 478 patients, the most common cause of tinnitus was NIHL (37.8%) (Tyler, 2000).The primary role of the physician is prevention and early detection, and it should be noted that many of the hazardous noises, are not due to job (Cummings et al., 2005). Because tinnitus is of commonly referred complaints to ENT clinics, and due to its inability to cure in the present time, it is hoped that this research as a basis for further studies on the epidemiology of the more common causes of tinnitus have been set up to solve social problems and to provide appropriate solutions and to treat or at least take steps to prevent it. The purpose of this study was to determine the causes of tinnitus in patients referred to ENT Clinic of Imam Khomeini Hospital, Urmia in 2012-2013. A study in 2007 was performed in Portugal to evaluate the clinical course and audiometric of the people who underwent chemotherapy with cisplatin. In this study, four patients (females), 38 to 69 years participated. For them speech and tonal audiometry were performed before and after treatment with cisplatin. One person showed no hearing impairment, mild to moderate bilateral hearing loss at other three persons in frequencies KHz8-6 was created and tinnitus was developed after chemotherapy. In this research it is recommended that all patients who are undergoing chemotherapy with cisplatin should be evaluated for early detection of hearing loss by audiometry (Mota et al., 2007).

In a study in 2010 with the aim of estimating the prevalence of auditory cues and to estimate the prevalence of exposure to hearing damage causes and analyze the relationship among these factors in a group of healthy young men were studied. 839 males, 22-19 years old, participated in this study. Data were collected by questionnaires and audiometry. The prevalence of tinnitus, sensitivity to sound and hearing defects were 32.2%, 15.5% and 14.5%, respectively. Contact with noise in 21.4% and loud music in 15.5% of participants were reported. People who had experienced tinnitus after exposure to noise was at higher risk for hearing loss in high frequencies, tinnitus and noise sensitivity. Those who listened to loud music had a higher prevalence of tinnitus but no hearing impairment (Muhr & Rosenhall, 2010). A study in Italy in 2005 was performed to determine the prevalence of tinnitus in patients with hypertension and the effects of different antihypertensive drugs on the incidence of tinnitus in these patients. This prospective single-blind study was performed in patients aged 75-18 years with uncontrolled hypertension that were receiving antihypertensive treatment. 476 patients participated in the study (283 males and 193 females), 84 patients (17.6%) cited occasionally or lasted buzz. The incidence of tinnitus was significantly higher in patients who received diuretics (72 out of 265 patients (27.2%) (Borghi et al., 2005). In 2003, a study was performed in Polish aimed to estimate the incidence of tinnitus caused by acoustic neuroma. Of the 2600 patients evaluated, 10 patients (0.34%) had schwannomas and stybolar (Raj-Koziak et al., 2003). A study in 2010 in America was performed to determine the incidence and relative risk of buzzing due to ototoxic drugs. Prospective study was performed to assess significant changes in audiology of 488 soldiers that were carried out chemotherapy drugs, ototoxic antibiotics or non-ototoxic medicines (as control group). The initial amount of buzz (compared to the general population of the same age) was higher (47%). People who were taking ototoxic medicine had significantly higher risk to create a buzz; that this risk was the highest for chemotherapy. Cisplatin increase the risk 5.53-fold and carboplatin 3.75-fold. Ototoxic antibiotics have borderline risks (2.81) to create a buzz. Unlike other studies, in this study individual factors (aging or hearing loss) or treatment (days of consumption or cumulative dose) did not affect the incidence of tinnitus during treatment (Dille et al., 2010). A study in Brazil in 2009 was performed to investigate and describe the buzz of the elderly. For this purpose, a questionnaire containing tinnitus and its impact on the life of the patient and the disease history was completed by 100 people. 61% of participants were female and the mean age was 69 years, 76% of non-pulsatile tinnitus and 57% were bilateral. No association was found between the degree of hearing loss and tinnitus patient dissatisfaction. Most of the findings in audiometric tests were presbycusis (Ferreira et al., 2009).

## 2. Materials and Methods

This is a cross-sectional analytic study on 184 patients with tinnitus referred to ENT clinic of Imam Khomeini Hospital, Urmia. The clinical examination including otoscopy was performed by a physician and the patient’s pure-tone audiometry and speech was performed by 822 Madsen OB audiometers, then the questionnaire that contains information such as the description of the profile and history of tinnitus, medical conditions and treatments as well as job history is completed. And any of their previous medical records were used to complete the questionnaire. For patients with pulsatile tinnitus for diagnosis of BIH, fundoscopy (see foot edema) and LP (CSF pressure above mmH2O200) and for carotid artery Doppler ultrasonography for ACAD and in cases where tinnitus and hearing loss was unilateral or asymmetric (suspected CPA pathology) MRI of the brain was performed with and without injection. Finally, by collecting information based on clinical findings and other assessments the cause of patient’s tinnitus was determined and treated according to the cause. Data used for this study were collected using a questionnaire and audiometry. And finally, after collecting data diagrams and frequency was used to answer research questions. Also to respond the hypothesis, the Chi-square test for comparison of qualitative variables and ANOVA test for comparison of mean hearing loss in terms of dB in groups were studied. For the above analysis SPSS16 software was used. (Dalfard & Mohammadi, 2012)

## 3. Results

This is a cross-sectional analytic study that was conducted on 184 patients with tinnitus referred to ENT clinic of Imam Khomeini Hospital, Urmia during the six months during 2012-2013. The study included 111 men and 73 women, mean age 50.6 years, age range 18 to 90 years. (Tables 1A and 1B)

**Table 1A:** Tinnitus frequency distribution according to gender

Relative frequency	Absolute frequency	Gender
60.3	111	Male
39.7	73	Female

**Table 1B:** The age frequency distribution of patients with tinnitus

Frequency	Aga
3	<20
20	20-29
32	30-39
36	40-49
30	50-59
29	60-69
22	70-79
11	80-89
1	>90

Of the patients, 168 patients (91.3%) had non-pulsatile tinnitus and 16 patients had pulsatile tinnitus. (Table 2)

**Table 2:** Frequency distribution of tinnitus on quality

Relative frequency	Absolute frequency	Tinnitus quality
91.3	168	Non-pulsatile
8.7	16	Pulsatile

The most buzzing area was the left ear with 35.9% and the lowest percentage was feeling the buzz in head with 7%. (Table 3)

**Table 3:** Tinnitus frequency distribution based on its position

Relative frequency	Absolute frequency	Area of tinnitus
28.2	52	Right ear
35.9	66	Left ear
28.8	53	Both ears
7	13	Head

100	184	Total

Average hearing loss of dB patients was 31.4%, 79.9% had sensorineural hearing loss and 50.5% had hearing loss in the high frequencies. There were not hearing loss in 23 cases. Remarkably, there was a deaf person with tinnitus. 12.5% of patients showed no hearing loss in audiometry and 42.9% of patients showed normal hearing loss (less than dB 25). (Tables 4, 5 and 6)

**Table 4:** Frequency distribution of types of hearing loss of patients with tinnitus

Relative frequency	Absolute frequency	Type of hearing loss
79.9	147	Sensorineural
1.1	2	Transitional
6	11	Mixed
12.5	23	Without hearing loss
0.5	1	Deaf

100	184	Total

**Table 5:** Frequency distribution of hearing loss frequency in patients with tinnitus

50.5	93	High frequencies

3.3	6	Low frequencies

33.7	62	All frequencies

12.5	23	Without hearing loss

100	184	Total

**Table 6:** Frequency distribution of hearing loss intensity in patients with tinnitus

Relative frequency	Absolute frequency	Intensity of hearing loss
42.9	79	Normal
21.2	39	Mild
17.4	32	Average
7.1	13	Moderate to severe
8.7	16	Intense
2.7	5	Deep

100	184	Total

Each patient’s tinnitus after examination, audiometry and questionnaire, which contains information such as the description of the profile and history of tinnitus, medical conditions and treatments as well as occupational exposure history, was determined. In total, 90.2% of patients were diagnosed and for 18 patients (9.8%) containing 16 patients with non-pulsatile tinnitus and 2 patients with pulsatile tinnitus did not find any known cause. Most causes of tinnitus were noise, including the noise of work, military or history of being in front and recreation noise (19.6%), ototoxicity (16.8%) and presbycusis (16.3%). Ear infections are also made up 9.8% of patients that the chronic central otitis was accounted for half of the cases. Causes of pulsatile tinnitus include glomus tumor, Eustachian tube dysfunction, and malformations of arterial venous of the head and neck. In all of the causes examined in any compact cerumen disease, neurocifilis, and palatal myoclonus were not diagnosed as causes of tinnitus (Tables 7 and 8).

**Table 7:** Frequency of non-pulsatile tinnitus causes

Real percentage	Relative frequency		Absolute frequency		Causes of pulsatile tinnitus		
21.4		12.5		23	Occupational	Noise

	19.6	5.5	36	10	Military	

		1.6		3	Recreational	

7.1	6.5		12		Acoustic trauma	

18.5	16.8		31		Ototoxicity	

17.9	16.3		30		Presbycusis	

14.9		3.8		7	Head damage (TBI)	Other diseases

		4.9		9	Meniere	

	13.6	1.6	25	3	Schwannomas and Stybolar	

		1.6		3	Otoscleroses	

		0		0	Compact cerumen	

		1.6		3	MS	

10.7		0.5		1	External otitis	Ear infections

		4.9		9	Central chronic otitis	

		0.5		1	Labyrinth	

	9.8	0.5	18	1	Herpes zoster	

		1.1		2	Measles	

		2.2		4	Orion	

		0		0	Neurosifilis	

9.5	8.7		16		Other Causes	

100	91.3		168		Total	

**Table 8:** Frequency of pulsatile tinnitus causes

Real percentage	Relative frequency	Absolute frequency	Causes of pulsatile tinnitus
6.25	0.5	1	BIH
6.25	0.5	1	ACAD
25	2.2	4	Glomus tumors
25	2.2	4	AVM/AVF
0	0	0	Palatal myoclonus
25	2.2	4	Eustachian tube dysfunction
12.5	1.1	2	Other causes

100	8.7	16	Total

Noise with 31.5% and presbycusis with 13.5% showed up most causes of tinnitus in males. Also, in females ototoxicity with 27.4% and presbycusis with 20.6% were the causes of tinnitus. Figure 11 shows frequency of causes of tinnitus in patients by gender. In terms of noise 35 of 36 cases were male and 26 patients had a loss in the high frequencies. Noise caused 43.7 dB hearing loss in high frequencies. Acoustic trauma in 11 of 12 cases loss was just at high frequencies with the mean of 35.8dB. Ototoxicity and presbycusis also caused 49.8 and 57.5dB loss at high frequencies, respectively. There was a significant relationship between age and hearing loss of the tinnitus patients. (P-value<0.001 of R=0.315), meaning that the prevalence of hearing loss increases with age. But there was no significant difference between sexes and hearing loss. That is, the average hearing loss in men was 31.9dB and in women was 30.2dB. Between the degree of hearing loss and tinnitus quality, there was a significant relationship (P-value=0.002 and t-test=3.198). This means that the people who had non-pulsatile tinnitus the prevalence of hearing loss was higher than the people who had pulsatile tinnitus (32.9dB versus 13.1dB). Also there is a significant relationship with the reliance index of 95% between hearing loss and the cause of tinnitus (P-value<0.001 and F=4.327), so that the highest average cases of hearing loss was otoscleroses (70dB) and ear infections (49.2dB) and the lowest cases of hearing loss was pulsatile tinnitus (13.1dB) and acoustic trauma (17.5dB).

## 4. Discussion

According to previous studies, the main causes of tinnitus are NIHL, presbycusis and ototoxicity (Cummings et al., 2005; Bauer & Brozoski, 2008). Depending on the population studied, NIHL forms from about 15% to 80% of tinnitus causes (Tyler, 2000). In 1960, in Reed study 200 patients with tinnitus and were examined in ENT clinic. Cause of 15.5% of patients was noise. Also in Axelsson and Barrenas study in 1991 of 411 patients, 33% of patients and in the Meikle Taylor study in 1984 from 1860 patients the cause of tinnitus was noise in 80% of males and in 31% of females. In this study, 20% of the cases belonged to the noise that is consistent with the results of Reed study. Our research revealed that there is no study similar to this study in Iran. In this study, 61% were males and 36% of cases had bilateral tinnitus and 80% had SNHL that was similar to the results of Madani and Mohammadi in 1998 in Bu Alisina hospital. Also, 18% of tinnitus was due to contact noise (factory work or engage in war) that is consistent with the results of our study. In both of these studies, it was shown that male gender and occupational status increases the frequency of buzz (Madani Syed Abdullah Mohammad Kebri, 2001). Previous findings have shown that about 10% of tinnitus occurs in the absence of specific hearing pathology in routine clinical testing (Bauer et al., 2008). In this study, 12.5% of patients showed lack of fall in the hearing. Also similar to Palmer study in 2002 in the UK, the rate of patients with age-related hearing loss, the prevalence of hearing loss increases with age (Palmer et al., 2002). In the 15-year study of Sismanis on 145 patients with pulsatile tinnitus, the most common reasons include BIH (39%), ACAD (17%) and Glomus tumors (12%). In this study there was the lack of diagnosis in 9% of cases (Sismanis, 1998). In our study, Glomus tumor, Eustachian tube dysfunction and AVM/AVF (25% each) were the most common causes, the two latter were accounted lower rates in Sismanis study that is perhaps due to the lower sample size in our study or due to genetic differences. In Raj study in 2003, Poland 0.3% of the tinittus causes was because of schwannoma and stybolar (Koziak et al., 2003). In this study, 1.6% of patients had schwannomas and stybolar. Valianatou study in Greece in 2001 from 80 cases in 27 patients (34%) had tinnitus associated with acoustic trauma, 23% NIHL, 6% presbycusis, 5% otoscleroses, 2.5% head damage (TBI) and 15% idiopathic cases that compared to our study acoustic trauma was more frequent. Common and preventable causes of tinnitus are noise, acoustic trauma, and ototoxic drugs that have total of 43% of all cases in this study. NIHL is completely preventable injury that except by accident can reduce the noise level at the source and by use of ear protection it can be entirely avoided. Exposure to noise can cause hearing loss without tinnitus and considering that, buzz can cause more disability than hearing loss, bring about behavioral and social problems, it is necessary to consider hearing protection programs.

While the deleterious effects of continuous noise and percussion in the military and battlefield environments on public health, including the health of hearing devices are well known, harmful effects of noise pollution on human health is so serious that warnings regarding the daily noise exposure including traffic, noise at work and listen to music at high intensity levels are raised; most social groups due to lack of adequate knowledge cannot understand that long listening to loud music cause hearing loss and tinnitus. We all know that young workers often simply do not realize the consequences of such a fall in the hearing that we can show tinnitus and hearing loss in terms of acoustic to population at risk to have a better understanding and awareness of the problems and motivated them to use ear protection when noise increases. By raising the level of awareness, providing information and research results to medical centers and create jobs and employment protection, deleterious effects caused by noise can be prevented. Thus, one of the useful measures will be widespread and public education. Irrational prescribing of ototoxic drugs without indication especially antibiotics, aspirin and NSAIDs (the most common drugs used in this study were ototoxic) either outpatient or inpatient departments increased incidence of tinnitus that with rational prescribing of these drugs for patients can reduce the prevalence of tinnitus that cause sleep disorders and concentration problems, depression and anxiety, and help the public health community. Also it is recommended for patients who are treated with ototoxic drugs, evaluated for early detection of hearing loss by audiometry. Although NIHL is the most common cause of tinnitus but little research has been done in this area. Because people with NIHL were almost all male, aged 20 to 60 years there are suitable possibilities for epidemiological studies. These studies have guidelines for solving this annoying and more or less untreatable problem.
